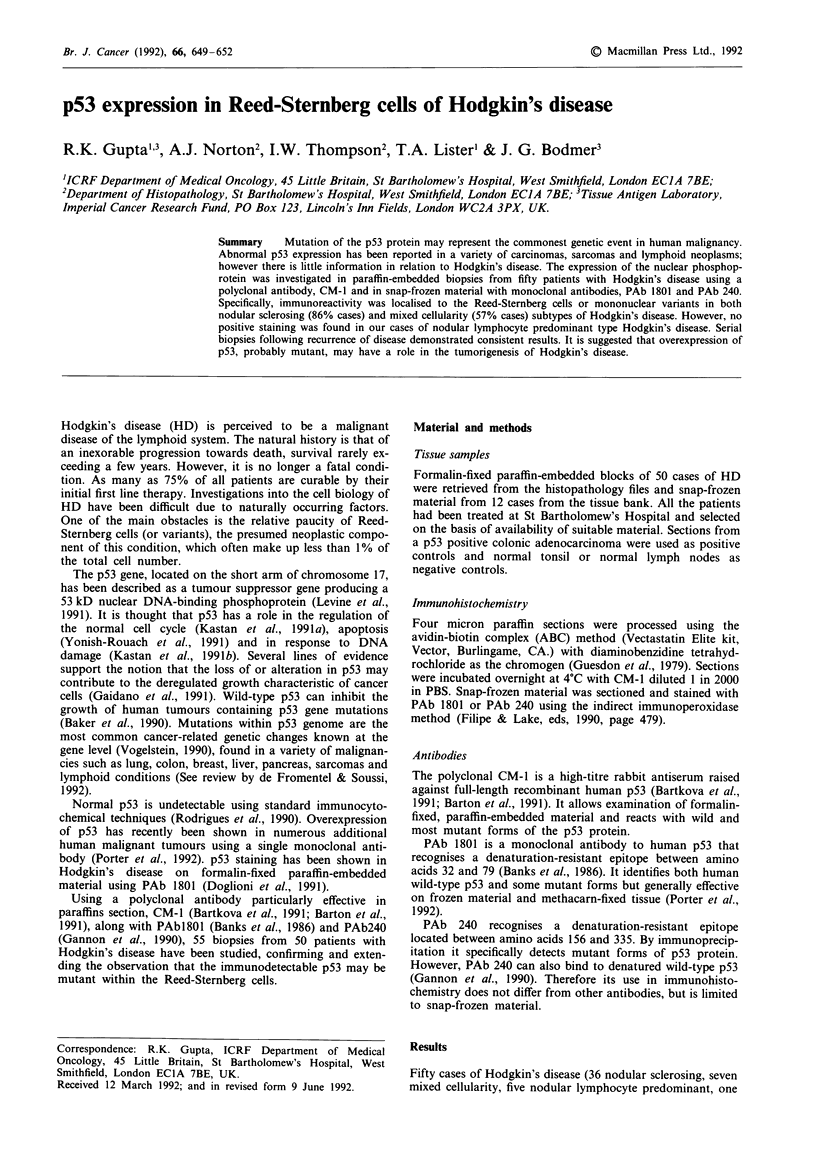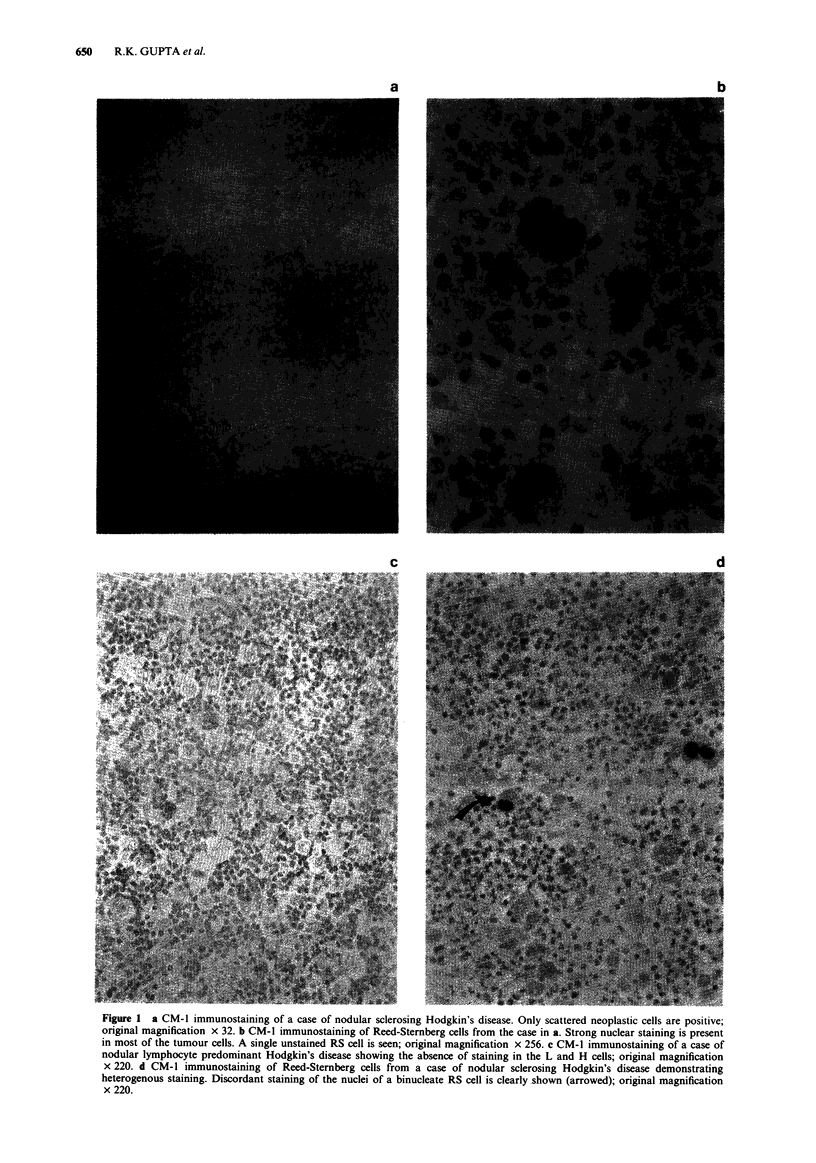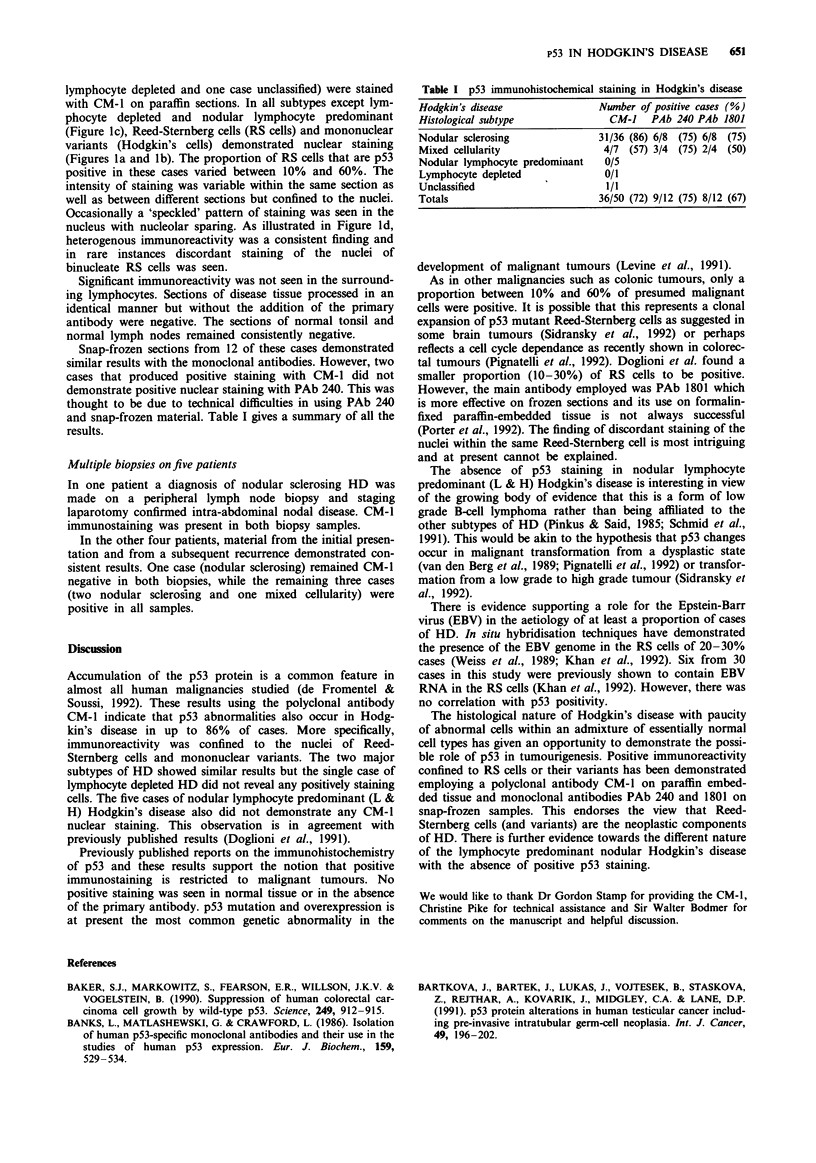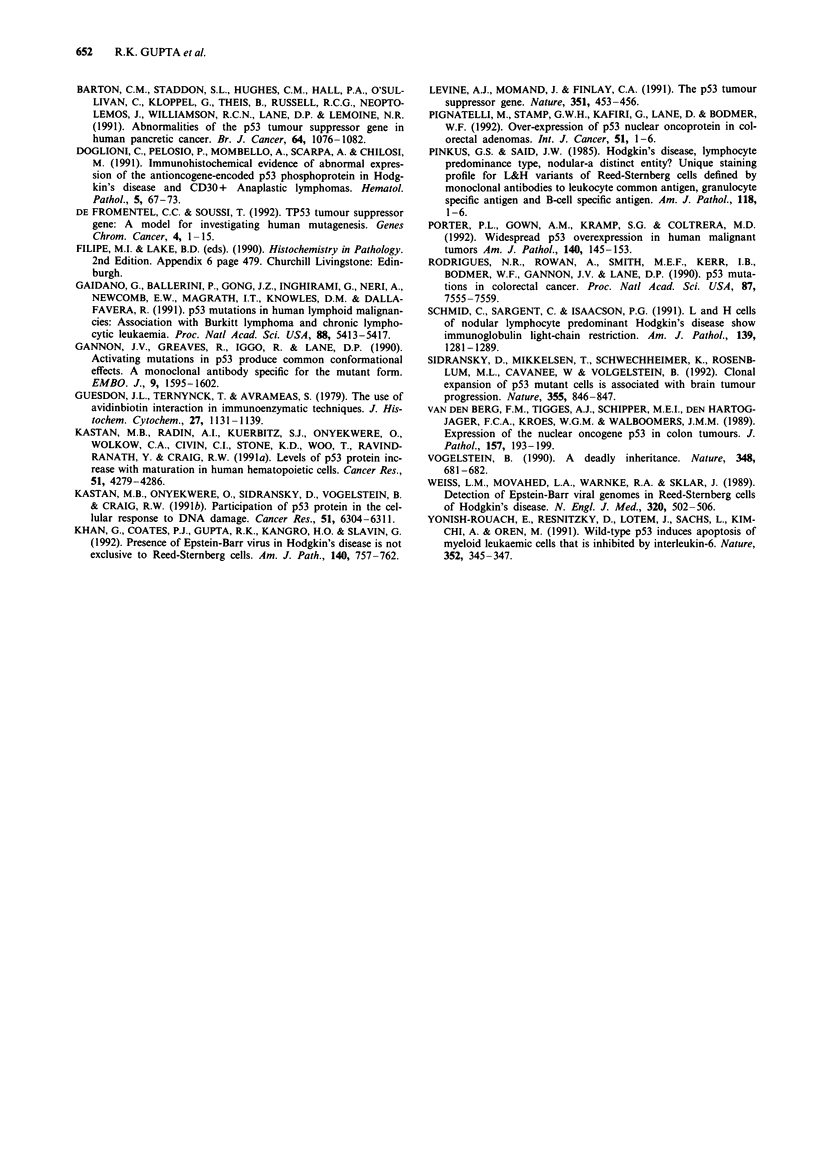# p53 expression in Reed-Sternberg cells of Hodgkin's disease.

**DOI:** 10.1038/bjc.1992.331

**Published:** 1992-10

**Authors:** R. K. Gupta, A. J. Norton, I. W. Thompson, T. A. Lister, J. G. Bodmer

**Affiliations:** ICRF Department of Medical Oncology, St Bartholomew's Hospital, West Smithfield, London, UK.

## Abstract

**Images:**


Br. J. Cancer (1992), 66, 649-652 © Macmillan Press Ltd., 1992

### p53 expression in Reed-Sternberg cells of Hodgkin's disease

R.K. Gupta[1,3], A.J. Norton[2], I.W. Thompson[2], T.A. Lister[1] & J. G. Bodmer[3]

[1]*ICRF Department of Medical Oncology, 45 Little Britain, St Bartholomew's Hospital, West Smithfield, London EC1A 7BE;* [2]*Department of Histopathology, St Bartholomew's Hospital, West Smithfield, London EC1A 7BE;* [3]*Tissue Antigen Laboratory, Imperial Cancer Research Fund, PO Box 123, Lincoln's Inn Fields, London WC2A 3PX, UK.*

**Summary** Mutation of the p53 protein may represent the commonest genetic event in human malignancy. Abnormal p53 expression has been reported in a variety of carcinomas, sarcomas and lymphoid neoplasms; however there is little information in relation to Hodgkin's disease. The expression of the nuclear phosphoprotein was investigated in paraffin-embedded biopsies from fifty patients with Hodgkin's disease using a polyclonal antibody, CM-1 and in snap-frozen material with monoclonal antibodies, PAb 1801 and PAb 240. Specifically, immunoreactivity was localised to the Reed-Sternberg cells or mononuclear variants in both nodular sclerosing (86% cases) and mixed cellularity (57% cases) subtypes of Hodgkin's disease. However, no positive staining was found in our cases of nodular lymphocyte predominant type Hodgkin's disease. Serial biopsies following recurrence of disease demonstrated consistent results. It is suggested that overexpression of p53, probably mutant, may have a role in the tumorigenesis of Hodgkin's disease.

Hodgkin's disease (HD) is perceived to be a malignant disease of the lymphoid system. The natural history is that of an inexorable progression towards death, survival rarely exceeding a few years. However, it is no longer a fatal condition. As many as 75% of all patients are curable by their initial first line therapy. Investigations into the cell biology of HD have been difficult due to naturally occurring factors. One of the main obstacles is the relative paucity of Reed-Sternberg cells (or variants), the presumed neoplastic component of this condition, which often make up less than 1% of the total cell number.

The p53 gene, located on the short arm of chromosome 17, has been described as a tumour suppressor gene producing a 53 kD nuclear DNA-binding phosphoprotein (Levine *et al.*, 1991). It is thought that p53 has a role in the regulation of the normal cell cycle (Kastan *et al.*, 1991*a*), apoptosis (Yonish-Rouach *et al.*, 1991) and in response to DNA damage (Kastan *et al.*, 1991*b*). Several lines of evidence support the notion that the loss of or alteration in p53 may contribute to the deregulated growth characteristic of cancer cells (Gaidano *et al.*, 1991). Wild-type p53 can inhibit the growth of human tumours containing p53 gene mutations (Baker *et al.*, 1990). Mutations within p53 genome are the most common cancer-related genetic changes known at the gene level (Vogelstein, 1990), found in a variety of malignancies such as lung, colon, breast, liver, pancreas, sarcomas and lymphoid conditions (See review by de Fromentel & Soussi, 1992).

Normal p53 is undetectable using standard immunocytochemical techniques (Rodrigues *et al.*, 1990). Overexpression of p53 has recently been shown in numerous additional human malignant tumours using a single monoclonal antibody (Porter *et al.*, 1992). p53 staining has been shown in Hodgkin's disease on formalin-fixed paraffin-embedded material using PAb 1801 (Doglioni *et al.*, 1991).

Using a polyclonal antibody particularly effective in paraffins section, CM-1 (Bartkova *et al.*, 1991; Barton *et al.*, 1991), along with PAb1801 (Banks *et al.*, 1986) and PAb240 (Gannon *et al.*, 1990), 55 biopsies from 50 patients with Hodgkin's disease have been studied, confirming and extending the observation that the immunodetectable p53 may be mutant within the Reed-Sternberg cells.

Correspondence: R.K. Gupta, ICRF Department of Medical Oncology, 45 Little Britain, St Bartholomew's Hospital, West Smithfield, London EC1A 7BE, UK.
Received 12 March 1992; and in revised form 9 June 1992.

### Material and methods

### *Tissue samples*

Formalin-fixed paraffin-embedded blocks of 50 cases of HD were retrieved from the histopathology files and snap-frozen material from 12 cases from the tissue bank. All the patients had been treated at St Bartholomew's Hospital and selected on the basis of availability of suitable material. Sections from a p53 positive colonic adenocarcinoma were used as positive controls and normal tonsil or normal lymph nodes as negative controls.

### *Immunohistochemistry*

Four micron paraffin sections were processed using the avidin-biotin complex (ABC) method (Vectastatin Elite kit, Vector, Burlingame, CA.) with diaminobenzidine tetrahydrochloride as the chromogen (Guesdon *et al.*, 1979). Sections were incubated overnight at 4°C with CM-1 diluted 1 in 2000 in PBS. Snap-frozen material was sectioned and stained with PAb 1801 or PAb 240 using the indirect immunoperoxidase method (Filipe & Lake, eds, 1990, page 479).

### *Antibodies*

The polyclonal CM-1 is a high-titre rabbit antiserum raised against full-length recombinant human p53 (Bartkova *et al.*, 1991; Barton *et al.*, 1991). It allows examination of formalin-fixed, paraffin-embedded material and reacts with wild and most mutant forms of the p53 protein.

PAb 1801 is a monoclonal antibody to human p53 that recognises a denaturation-resistant epitope between amino acids 32 and 79 (Banks *et al.*, 1986). It identifies both human wild-type p53 and some mutant forms but generally effective on frozen material and methacarn-fixed tissue (Porter *et al.*, 1992).

PAb 240 recognises a denaturation-resistant epitope located between amino acids 156 and 335. By immunoprecipitation it specifically detects mutant forms of p53 protein. However, PAb 240 can also bind to denatured wild-type p53 (Gannon *et al.*, 1990). Therefore its use in immunohistochemistry does not differ from other antibodies, but is limited to snap-frozen material.

### Results

Fifty cases of Hodgkin's disease (36 nodular sclerosing, seven mixed cellularity, five nodular lymphocyte predominant, one

**Figure 1** **a** CM-1 immunostaining of a case of nodular sclerosing Hodgkin's disease. Only scattered neoplastic cells are positive; original magnification × 32. **b** CM-1 immunostaining of Reed-Sternberg cells from the case in **a**. Strong nuclear staining is present in most of the tumour cells. A single unstained RS cell is seen; original magnification × 256. **c** CM-1 immunostaining of a case of nodular lymphocyte predominant Hodgkin's disease showing the absence of staining in the L and H cells; original magnification × 220. **d** CM-1 immunostaining of Reed-Sternberg cells from a case of nodular sclerosing Hodgkin's disease demonstrating heterogenous staining. Discordant staining of the nuclei of a binucleate RS cell is clearly shown (arrowed); original magnification × 220.

lymphocyte depleted and one case unclassified) were stained with CM-1 on paraffin sections. In all subtypes except lymphocyte depleted and nodular lymphocyte predominant (Figure 1c), Reed-Sternberg cells (RS cells) and mononuclear variants (Hodgkin's cells) demonstrated nuclear staining (Figures 1a and 1b). The proportion of RS cells that are p53 positive in these cases varied between 10% and 60%. The intensity of staining was variable within the same section as well as between different sections but confined to the nuclei. Occasionally a 'speckled' pattern of staining was seen in the nucleus with nucleolar sparing. As illustrated in Figure 1d, heterogenous immunoreactivity was a consistent finding and in rare instances discordant staining of the nuclei of binucleate RS cells was seen.

Significant immunoreactivity was not seen in the surrounding lymphocytes. Sections of disease tissue processed in an identical manner but without the addition of the primary antibody were negative. The sections of normal tonsil and normal lymph nodes remained consistently negative.

Snap-frozen sections from 12 of these cases demonstrated similar results with the monoclonal antibodies. However, two cases that produced positive staining with CM-1 did not demonstrate positive nuclear staining with PAb 240. This was thought to be due to technical difficulties in using PAb 240 and snap-frozen material. Table I gives a summary of all the results.

*Multiple biopsies on five patients*

In one patient a diagnosis of nodular sclerosing HD was made on a peripheral lymph node biopsy and staging laparotomy confirmed intra-abdominal nodal disease. CM-1 immunostaining was present in both biopsy samples.

In the other four patients, material from the initial presentation and from a subsequent recurrence demonstrated consistent results. One case (nodular sclerosing) remained CM-1 negative in both biopsies, while the remaining three cases (two nodular sclerosing and one mixed cellularity) were positive in all samples.

### Discussion

Accumulation of the p53 protein is a common feature in almost all human malignancies studied (de Fromentel & Soussi, 1992). These results using the polyclonal antibody CM-1 indicate that p53 abnormalities also occur in Hodgkin's disease in up to 86% of cases. More specifically, immunoreactivity was confined to the nuclei of Reed-Sternberg cells and mononuclear variants. The two major subtypes of HD showed similar results but the single case of lymphocyte depleted HD did not reveal any positively staining cells. The five cases of nodular lymphocyte predominant (L & H) Hodgkin's disease also did not demonstrate any CM-1 nuclear staining. This observation is in agreement with previously published results (Doglioni *et al.*, 1991).

Previously published reports on the immunohistochemistry of p53 and these results support the notion that positive immunostaining is restricted to malignant tumours. No positive staining was seen in normal tissue or in the absence of the primary antibody. p53 mutation and overexpression is at present the most common genetic abnormality in the development of malignant tumours (Levine *et al.*, 1991).

**Table I** p53 immunohistochemical staining in Hodgkin's disease

| *Hodgkin's disease Histological subtype* | *Number of positive cases (%)* CM-1 | PAb 240 | PAb 1801 |
|---|---|---|---|
| Nodular sclerosing | 31/36 (86) | 6/8 (75) | 6/8 (75) |
| Mixed cellularity | 4/7 (57) | 3/4 (75) | 2/4 (50) |
| Nodular lymphocyte predominant | 0/5 | | |
| Lymphocyte depleted | 0/1 | | |
| Unclassified | 1/1 | | |
| Totals | 36/50 (72) | 9/12 (75) | 8/12 (67) |

As in other malignancies such as colonic tumours, only a proportion between 10% and 60% of presumed malignant cells were positive. It is possible that this represents a clonal expansion of p53 mutant Reed-Sternberg cells as suggested in some brain tumours (Sidransky *et al.*, 1992) or perhaps reflects a cell cycle dependance as recently shown in colorectal tumours (Pignatelli *et al.*, 1992). Doglioni *et al.* found a smaller proportion (10–30%) of RS cells to be positive. However, the main antibody employed was PAb 1801 which is more effective on frozen sections and its use on formalin-fixed paraffin-embedded tissue is not always successful (Porter *et al.*, 1992). The finding of discordant staining of the nuclei within the same Reed-Sternberg cell is most intriguing and at present cannot be explained.

The absence of p53 staining in nodular lymphocyte predominant (L & H) Hodgkin's disease is interesting in view of the growing body of evidence that this is a form of low grade B-cell lymphoma rather than being affiliated to the other subtypes of HD (Pinkus & Said, 1985; Schmid *et al.*, 1991). This would be akin to the hypothesis that p53 changes occur in malignant transformation from a dysplastic state (van den Berg *et al.*, 1989; Pignatelli *et al.*, 1992) or transformation from a low grade to high grade tumour (Sidransky *et al.*, 1992).

There is evidence supporting a role for the Epstein-Barr virus (EBV) in the aetiology of at least a proportion of cases of HD. *In situ* hybridisation techniques have demonstrated the presence of the EBV genome in the RS cells of 20–30% cases (Weiss *et al.*, 1989; Khan *et al.*, 1992). Six from 30 cases in this study were previously shown to contain EBV RNA in the RS cells (Khan *et al.*, 1992). However, there was no correlation with p53 positivity.

The histological nature of Hodgkin's disease with paucity of abnormal cells within an admixture of essentially normal cell types has given an opportunity to demonstrate the possible role of p53 in tumourigenesis. Positive immunoreactivity confined to RS cells or their variants has been demonstrated employing a polyclonal antibody CM-1 on paraffin embedded tissue and monoclonal antibodies PAb 240 and 1801 on snap-frozen samples. This endorses the view that Reed-Sternberg cells (and variants) are the neoplastic components of HD. There is further evidence towards the different nature of the lymphocyte predominant nodular Hodgkin's disease with the absence of positive p53 staining.

We would like to thank Dr Gordon Stamp for providing the CM-1, Christine Pike for technical assistance and Sir Walter Bodmer for comments on the manuscript and helpful discussion.

### References

BAKER, S.J., MARKOWITZ, S., FEARSON, E.R., WILLSON, J.K.V. & VOGELSTEIN, B. (1990). Suppression of human colorectal carcinoma cell growth by wild-type p53. *Science*, **249**, 912–915.

BANKS, L., MATLASHEWSKI, G. & CRAWFORD, L. (1986). Isolation of human p53-specific monoclonal antibodies and their use in the studies of human p53 expression. *Eur. J. Biochem.*, **159**, 529–534.

BARTKOVA, J., BARTEK, J., LUKAS, J., VOJTESEK, B., STASKOVA, Z., REJTHAR, A., KOVARIK, J., MIDGLEY, C.A. & LANE, D.P. (1991). p53 protein alterations in human testicular cancer including pre-invasive intratubular germ-cell neoplasia. *Int. J. Cancer*, **49**, 196–202.

BARTON, C.M., STADDON, S.L., HUGHES, C.M., HALL, P.A., O'SULLIVAN, C., KLOPPEL, G., THEIS, B., RUSSELL, R.C.G., NEOPTOLEMOS, J., WILLIAMSON, R.C.N., LANE, D.P. & LEMOINE, N.R. (1991). Abnormalities of the p53 tumour suppressor gene in human pancretic cancer. *Br. J. Cancer*, **64**, 1076-1082.

DOGLIONI, C., PELOSIO, P., MOMBELLO, A., SCARPA, A. & CHILOSI, M. (1991). Immunohistochemical evidence of abnormal expression of the antioncogene-encoded p53 phosphoprotein in Hodgkin's disease and CD30+ Anaplastic lymphomas. *Hematol. Pathol.*, **5**, 67-73.

DE FROMENTEL, C.C. & SOUSSI, T. (1992). TP53 tumour suppressor gene: A model for investigating human mutagenesis. *Genes Chrom. Cancer*, **4**, 1-15.

FILIPE, M.I. & LAKE, B.D. (eds). (1990). *Histochemistry in Pathology*. 2nd Edition. Appendix 6 page 479. Churchill Livingstone: Edinburgh.

GAIDANO, G., BALLERINI, P., GONG, J.Z., INGHIRAMI, G., NERI, A., NEWCOMB, E.W., MAGRATH, I.T., KNOWLES, D.M. & DALLA-FAVERA, R. (1991). p53 mutations in human lymphoid malignancies: Association with Burkitt lymphoma and chronic lymphocytic leukaemia. *Proc. Natl Acad. Sci. USA*, **88**, 5413-5417.

GANNON, J.V., GREAVES, R., IGGO, R. & LANE, D.P. (1990). Activating mutations in p53 produce common conformational effects. A monoclonal antibody specific for the mutant form. *EMBO. J.*, **9**, 1595-1602.

GUESDON, J.L., TERNYNCK, T. & AVRAMEAS, S. (1979). The use of avidinbiotin interaction in immunoenzymatic techniques. *J. Histochem. Cytochem.*, **27**, 1131-1139.

KASTAN, M.B., RADIN, A.I., KUERBITZ, S.J., ONYEKWERE, O., WOLKOW, C.A., CIVIN, C.I., STONE, K.D., WOO, T., RAVINDRANATH, Y. & CRAIG, R.W. (1991*a*). Levels of p53 protein increase with maturation in human hematopoietic cells. *Cancer Res.*, **51**, 4279-4286.

KASTAN, M.B., ONYEKWERE, O., SIDRANSKY, D., VOGELSTEIN, B. & CRAIG, R.W. (1991*b*). Participation of p53 protein in the cellular response to DNA damage. *Cancer Res.*, **51**, 6304-6311.

KHAN, G., COATES, P.J., GUPTA, R.K., KANGRO, H.O. & SLAVIN, G. (1992). Presence of Epstein-Barr virus in Hodgkin's disease is not exclusive to Reed-Sternberg cells. *Am. J. Path.*, **140**, 757-762.

LEVINE, A.J., MOMAND, J. & FINLAY, C.A. (1991). The p53 tumour suppressor gene. *Nature*, **351**, 453-456.

PIGNATELLI, M., STAMP, G.W.H., KAFIRI, G., LANE, D. & BODMER, W.F. (1992). Over-expression of p53 nuclear oncoprotein in colorectal adenomas. *Int. J. Cancer*, **51**, 1-6.

PINKUS, G.S. & SAID, J.W. (1985). Hodgkin's disease, lymphocyte predominance type, nodular-a distinct entity? Unique staining profile for L&H variants of Reed-Sternberg cells defined by monoclonal antibodies to leukocyte common antigen, granulocyte specific antigen and B-cell specific antigen. *Am. J. Pathol.*, **118**, 1-6.

PORTER, P.L., GOWN, A.M., KRAMP, S.G. & COLTRERA, M.D. (1992). Widespread p53 overexpression in human malignant tumors *Am. J. Pathol.*, **140**, 145-153.

RODRIGUES, N.R., ROWAN, A., SMITH, M.E.F., KERR, I.B., BODMER, W.F., GANNON, J.V. & LANE, D.P. (1990). p53 mutations in colorectal cancer. *Proc. Natl Acad. Sci. USA*, **87**, 7555-7559.

SCHMID, C., SARGENT, C. & ISAACSON, P.G. (1991). L and H cells of nodular lymphocyte predominant Hodgkin's disease show immunoglobulin light-chain restriction. *Am. J. Pathol.*, **139**, 1281-1289.

SIDRANSKY, D., MIKKELSEN, T., SCHWECHHEIMER, K., ROSENBLUM, M.L., CAVANEE, W & VOLGELSTEIN, B. (1992). Clonal expansion of p53 mutant cells is associated with brain tumour progression. *Nature*, **355**, 846-847.

VAN DEN BERG, F.M., TIGGES, A.J., SCHIPPER, M.E.I., DEN HARTOG-JAGER, F.C.A., KROES, W.G.M. & WALBOOMERS, J.M.M. (1989). Expression of the nuclear oncogene p53 in colon tumours. *J. Pathol.*, **157**, 193-199.

VOGELSTEIN, B. (1990). A deadly inheritance. *Nature*, **348**, 681-682.

WEISS, L.M., MOVAHED, L.A., WARNKE, R.A. & SKLAR, J. (1989). Detection of Epstein-Barr viral genomes in Reed-Sternberg cells of Hodgkin's disease. *N. Engl. J. Med.*, **320**, 502-506.

YONISH-ROUACH, E., RESNITZKY, D., LOTEM, J., SACHS, L., KIMCHI, A. & OREN, M. (1991). Wild-type p53 induces apoptosis of myeloid leukaemic cells that is inhibited by interleukin-6. *Nature*, **352**, 345-347.